# Coding of saliency by ensemble bursting in the amygdala of primates

**DOI:** 10.3389/fnbeh.2012.00038

**Published:** 2012-07-25

**Authors:** S. L. Gonzalez Andino, R. Grave de Peralta Menendez

**Affiliations:** Electrical Neuroimaging Group, Clinic of Neurology and Department of Neuroscience, University Medical Centre and Geneva University HospitalGeneva, Switzerland

**Keywords:** active vision, amygdala, ensemble bursting, fixations, saliency

## Abstract

Salient parts of a visual scene attract longer and earlier fixations of the eyes. Saliency is driven by bottom-up (image dependent) factors and top-down factors such as behavioral relevance, goals, and expertise. It is currently assumed that a saliency map defining eye fixation priorities is stored in neural structures that remain to be determined. Lesion studies support a role for the amygdala in detecting saliency. Here we show that neurons in the amygdala of primates fire differentially when the eyes approach to or fixate behaviorally relevant parts of visual scenes. Ensemble bursting in the amygdala accurately predicts main fixations during the free-viewing of natural images. However, fixation prediction is significantly better for faces—where a bottom-up computational saliency model fails—compared to unfamiliar objects and landscapes. On this basis we propose the amygdala as a locus for a saliency map and ensemble bursting as a saliency coding mechanism.

## Introduction

Vision is an active process during which gaze shift to fixate salient locations of the scene. Saliency is a task-dependent and dynamically changing concept: Red circular shapes are salient when looking for tomatoes but saliency is quickly reattributed to white objects when looking for mozzarella. However, neural mechanisms and structures implicated in defining saliency are not yet completely understood. A likely mechanism is that neurons that participate in coding specific visual stimuli are also involved in guiding the eyes to salient features of objects (Moore, 1999).

The amygdala is in an ideal position to detect visual saliency; it has reciprocal connections with multiple visually responsive areas in the temporal (Desimone and Gross, 1979; Amaral et al., 1992, 2003; Freese and Amaral, 2006) and frontal lobes (Ghashghaei and Barbas, 2002). It is composed by cells with large receptive fields that allow the localization of salient objects outside the foveated area (Rolls et al., 1994) and that show selective responses not only to faces, facial expressions and gaze direction (Rolls et al., 1994; Gothard et al., 2007; Hoffman et al., 2007; Rutishauser et al., 2011), but also to images with inherent or learned emotional significance (Gothard et al., 2007), what permits the amygdala to influence the way in which saliency is dynamically defined by the brain.

Based on the abnormal visual scanning of faces of patients with amygdala damage, Adolphs (Adolphs, 2008) suggested that the amygdala might act as a detector of “perceptual saliency and biological relevance” (Sander et al., 2005). Patients with schizophrenia (Sasson et al., 2007), social phobia (Horley et al., 2004), and autism (Adolphs et al., 2001), also shown “irregular” facial scanning patterns partially attributed to malfunctions of the amygdala (Grady and Keightley, 2002; Baron-Cohen, 2004). Importantly, salient elements of the scene elicit longer visual exploration and are generally fixated earlier (Henderson and Hollingworth, 1999). Thus, lack of an appropriate definition of saliency provided by the amygdala might explain the absence or reduction of fixations on novel objects observed in monkeys with amygdala lesions (Bagshaw et al., 1972) or the fixation impairments reported in humans (Adolphs et al., 2001, 2005) after amygdala damage.

Finally, brain areas responsible for top-down attentional effects are typically linked to oculomotor structures (Treue, 2003). Despite no reports of direct involvement of the amygdala in the planning and execution of eye movements, direct connections between the amygdala and subcortical oculomotor centers in the pons and midbrain (Han et al., 1997; Amaral et al., 2003) are well documented.

Thus, considering: (1) the visual selectivity of neurons in the amygdala to socially relevant signals, (2) the documented effects of lesions on eye's fixations, and (3) the opportunity of circuits in the amygdala to directly influence eye movements thanks to its connectivity, we hypothesized that either single or small populations of cells in the amygdala might differentially fire at behaviorally relevant locations of the visual scene to indicate saliency and help to choose where and for how long to fixate.

## Methods

**Figure 1:**
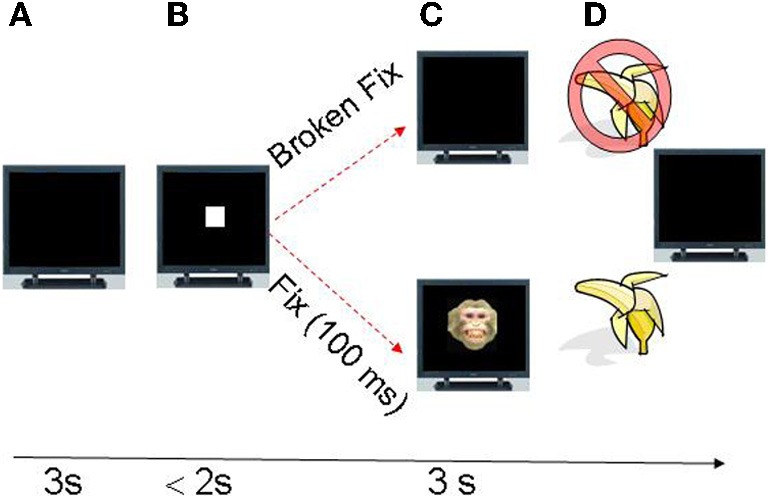
**Task progression. (A)** The trial starts with an empty monitor presented during three seconds (inter-trial period). **(B)** At the end of the inter-trial interval a fixation icon (white square) appeared in the center of the monitor and the monkey was required to make a saccade within 2 s to fixation area subtending three degrees of visual angle (dva) centered on the fixation icon (fixspot). **(C)** If the eyes remained fixated for 100ms, an image appeared which the monkey was allowed to freely view. The trial was aborted without reward if the monkey failed to fixate on the fixation icon for 100ms and the monitor became blank. **(D)** Juice reward was delivered if gaze was maintained within the boundary of the image for 3 s.

In the study (Figure 1) we obtained intraamygdaloid recordings from 263 cells in three monkeys allowed to freely scan full frequency color images depicting monkey faces with various facial expressions and gaze directions (averted or directed at the viewer) or non-faces (landscapes, abstract images, and objects). The horizontal and vertical eye position signals were simultaneously monitored using an infrared eye tracker.

The data were collected at the laboratory of K. Gothard were the experimental design, data collection and initial analysis, i.e., spike sorting took place. The electrode delivery system was a custom-built seven-channel Eckhorn drive (Thomas Recording). The system uses seven quartz glass coated, tungsten/platinum core electrodes, 80–100mm in diameter, and can deliver them to a depth of 25–35mm below the surface of the brain. The drive controls the depth of each electrode independently via precision motors which are used to expand or contract small the rubber tubes attached to the back of each electrode which serve to advance and withdraw the electrodes. The electrodes were delivered into the brain with 30-gauge sharp stainless steal cannulae that were driven 5mm into the brain, penetrating the dura. The target coordinates in the amygdala were calculated using the MRI-based method developed by (Saunders et al., 1990; Rebert et al., 1991; Zola-Morgan et al., 1991) and adapted to the amygdala by Amaral and colleagues (Amaral et al., 1992).

Built into the drive was a headstage amplifier (gain = 20) that directed signals to a Lynx-8 (Neuralynx, Tucson, AX) amplifier (gain = 2000). The sampling rate used for recording LFP data was 1000 Hz and was aquired using a Power 1401 data-acquisition system (Cambridge Electronics Design (CED), Cambridge, UK). Recorded data was stored for off-line analysis.

To begin the recording, the cannulae containing the electrodes were first advanced 5mm into the brain and the electrodes were then advanced to the MRI-determined steriotaxic coordinates of the target nuclei of the right amygdala. The electrodes were moved in small increments until a single unit or good signal to noise ratio was obtained. This was done so that the electrodes would be able to record single units as well as LFP data. The experiment was conducted over the course of several recording sessions spanning two years.

Off-line spike sorting relied on a template-matching algorithm (Spike2, CED) as described in (Gothard et al., 2007; Mosher et al., 2010).

### Subjects

Three (S, T, Q) adult male monkeys (*Macaca mulatta*) were used for intraamygdaloid recordings of neural activity. All procedures followed NIH guidelines for the use of non-human primates in biomedical research and were approved by the Institutional Animal Care and Use Committee of the University of Arizona.

Visual properties for the cells reported here were previously described in (Gothard et al., 2007) for monkey S and in (Mosher et al., 2010) for monkeys T and Q.

### Eye tracking and behavioral data acquisition

Horizontal and vertical eye position signals were monitored at 120 Hz using an infrared eye tracker (ISCAN, Burlington, MA). Scanpath data were recorded simultaneously with multiunit activity (20 KHz, frequency sampling), local field potentials (1000 Hz) and behavioral markers delivered to the data acquisition system by the CORTEX experimental control system (NIMH-supported freeware: info at http://www.cortex.salk.edu). The behavioral markers indicated the time of display and removal of the fixation icon, the time when fixation was achieved, and the time of display and removal of stimulus images.

### Behavioral task

Multiple single units (263) were recorded from three monkeys (S, T, and Q) trained on an image viewing task. During the task, the monkeys were head-restrained and seated in a primate chair 57 cm from the display monitor. A trial began with the display of a fixation icon, henceforth called the “fixspot”, which was a white square of 0.5 cm diameter (equivalent to 0.5° visual angle) (Figure 1). A saccade to the fixspot and a fixation of at least 100ms on it resulted in the removal of the fixspot and the display of a stimulus image (12 × 12° visual angle). Stimulus images were displayed for 3 s and were followed by a 3 s inter-trial interval (ITI). The stimuli (full frequency color images) depicted monkey faces with various facial expressions and gaze directions (averted or directed at the viewer) or objects (landscapes, abstract images, and random objects). The facial expressions included the following categories: (1) threatening faces (TH), (2) neutral faces (NE), (3) fearful faces (FG), and (4) appeasing faces (Lip smacking, LS). The monkeys were allowed to freely scan the image but had to maintain gaze within its boundaries. Successful fixation and image viewing were rewarded with fruit smoothie. Failure to fixate or to maintain gaze within image boundaries aborted the trial. On “error trials” in which the monkeys broke fixations before the required 100ms or fixate outside the fixspot boundaries, no image was presented and reward omitted.

### Statistical analysis of firing rates and categorization of neurons

For the analysis of the firing rates we relied on standard non-parametric tests as significant deviations from the normal distribution were often detected (Lilliefors test, *p* < 0.05). Cells were categorized as visually responsive, motor and visuo-motor. Visually responsive cells included cells that varied firing in response to the fixspot, the image, both or exclusively to some particular image categories (image selective cells). All the conclusions derived from the statistical analysis were further validated by the visual inspection of the raster plots and peri-event histograms.

Analysis windows of 200ms length (mean reaction time to complete fixations) were taken in the ITI period and the post-stimulus period. A neuron was classed as visually responsive when its mean firing rate after stimulus onset significantly changed (*p* < 0.01, paired non-parametric Wilcoxon signed rank test) with respect to the mean firing in the absence of visual stimulation (ITI). A neuron was classed as image selective when its mean firing in the post-image period was significantly different across the different image categories (*p* < 0.01, Kruskall–Wallis non-parametric one way ANOVA).

Finally, to evaluate motor related modulations in firing rates we took windows of four seconds length encompassing the one second preceding the presentation of the image, i.e., including the fixspot, and the three seconds of free image viewing. A neuron was classed as motor when its mean firing differed significantly between saccades and fixation periods (*p* < 0.01, non-paired, non-parametric Mann–Whitney *U*-tests).

Purely motor neurons were motor neurons not classed as visually responsive or image selective. Purely visual neurons were defined as visually responsive neurons for which mean firing remained unchanged by saccade/fixations. Visuo-motor neurons were neurons simultaneously classed as motor and visually selective.

### Detecting bursts and their surprise

The connectivity, selectivity of cells and fixation deficits following lesions to the amygdala (Bagshaw et al., 1972; Adolphs et al., 2005) suggest its involvement in defining saliency. Yet, the neural mechanisms used to signal saliency or the aspects of the visual scene marked as salient by the amygdala remain unknown. A likely electrophysiological correlate of target selection reflecting top-down saliency is the differential firing of the cells when eyes approach or fixate selected parts in an image.

A potential computational mechanism to signal saliency, already observed in the basal forebrain to indicate motivation (Lin and Nicolelis, 2008) or the supplementary eye field to indicate target selection (Hanes et al., 1995), is the ensemble bursting of neurons. We therefore investigated whether cells in the amygdala fire bursts at particularly salient image locations during the free image viewing period (three seconds). To detect bursts we relied on an algorithm previously used for cells in the supplementary eye field (Hanes et al., 1995).

For the analysis of the population activity during image viewing we used the spike burst detector based on Poisson spike train analysis described in Hanes et al., (Hanes et al., 1995) and Thompson et al., (Thompson et al., 1996). The analysis was done using the Matlab implementation of this algorithm available from the web site of the authors http://psych-s1.psy.vanderbilt.edu/faculty/schalljd/atools.php.

First, the Poisson spike train analysis was applied to each trial (three seconds image viewing and one second before image presentation) to identify periods of activity in which more spikes occurred than predicted from a Poisson random process having the overall average rate of the trial. In this analysis, we set the significance level α at 0.01. Second, a period was defined as a burst if containing at least four spikes. Third, the surprise of a burst was defined as *S* = -*logP* where *P* is the probability that a given time interval of length Δ*t* contains *n* or more spikes. Therefore, *S* is higher for the more unexpected bursts.

### Scanpaths analysis, labeled scanpaths and fixation hotspot map

Salient locations in a visual scene are fixated earlier and for longer times. Consequently, they can be identified by the appropriate analysis of the eye position data. The off-line analysis of eye position data was performed with the use of home-made matlab programs that identified and marked the onset and termination of each saccade using a velocity and acceleration threshold criteria in combination with a dwell time fixation detection (minimal fixation length set to 80ms). The duration of a fixation was defined as the time elapsed between two consecutive saccades. Fixation durations (and companion neural data) greater than 2000ms were also excluded. Each trial was inspected visually, and corrected if necessary.

For the image viewing period we computed the “Labeled Scanpath” and the Fixation Hotspot Map (FHM). The Labeled Scanpath is a three seconds length vector with label one assigned to saccades and label two to fixations. A summary labeled scanpath is the *N* times three seconds vector built by merging labeled scanpaths obtained for the *N* repetitions of the same image.

The FHM is a matrix with the same dimension as the originally presented image (300 × 300 pixels) that contains the number of fixations detected at each pixel during one image presentation. The FHM is convolved with a two dimensional Gaussian kernel with sigma of one degree of visual angle. A summary FHM is build by adding all individual Fixation Hotspot maps (FHMs) obtained for each presentation of the same image, normalized to reflect the percentage of total viewing time spent on fixations per pixel.

### Analysis of the scanpaths as a function of image category

To investigate the degree to which scanpaths vary as a function of the category of the image we relied on two different measures of scanpaths variability during the three seconds image viewing period. In this analysis, we excluded trials where monkeys brought the eyes outside the image boundaries. The images were divided into two large groups, Non-faces (NULL) and faces. Faces were further subdivided according to their emotional expression into four groups: (1) appeasing (LS), (2) threatening (TH), (3) fearful (FG), and (4) neutral (NE).

As a first measure we computed the percentage of time spent in fixations with respect to the total viewing time (three seconds). As a measure of the dispersion of fixations in each image, we calculated the perimeter of the convex hull enclosing all the fixations detected. The convex hull is the minimal convex set containing all the points. This measure was normalized, for each monkey, by the maximum perimeter observed within the NULL category.

### Evaluating predictions of a saliency model, and creating the amygdala (ASM) and the visual (VSM) saliency model

We hypothesized that if ensemble bursting within the amygdala is the mechanism signaling salient targets deserving detailed visual inspection then we should be able to infer the viewer's preferred fixations from the image locations at which ensemble bursts are detected. In other words, the bursts should exhibit spatial selectivity—a sort of saliency map—that coincides with pixels in the images that were repeatedly fixated across multiple image presentations or for longer periods.

To define pixels in the image that were repeatedly fixated we thresholded the Summary FHM to remove just the pixels receiving less than 20% of the maximum fixation time. Cutoff values between 10% and 30% lead to comparable results. For simplicity, we will continue to use henceforth the term Summary FHM to refer to this thresholded map.

The predictive power of a saliency model can be judged by computing some similarity measure between the saliency map created by the model and the summary FHM. To investigate if ensemble burst firing in the amygdala signals aspects in the scene driven by bottom-up or top-down saliency we compared the predictive value of two models: (1) a computational bottom-up visual saliency model (VSM) directly computed from the image features according to Itti, Koch and Neuber (Itti et al., 1998), and (2) an internally defined saliency model, that we called the amygdala saliency model (ASM) generated from the normalized ensemble bursting of small population of cells. The VSM model was computed for each image using the Matlab code freely available at: http://www.klab.caltech.edu/~harel/share/gbvs.php. Here we indistinctly denote by ASM and VSM the models and their associated maps.

Bursts were detected from the combined spiking activity of all simultaneously recorded neurons in a session. We created a 2D image from the ASM by assigning to each pixel in the trajectory of the eyes over which bursts were fired the surprise of the burst, i.e., a quantitative statistical measure of the improbability of the burst. Large values of surprise indicate significant increases in the rate of firing of the ensemble of cells. A summary ASM map was obtained by adding the surprise values for each repetition of the same image. Each pixel in the resultant image was divided by the total number of visits of the eyes it received to make the ASM independent from the time spent in fixations (normalized ensemble bursting).

The saliency models and the Summary FHM were compared using the area under the Receiver Operator characteristics (AuROC) curve to get a similarity measure bounded between zero and one. The ROC curve is the plot of the fraction of true positives (i.e., true positive rate TPR) vs. the fraction of false positives (i.e., false positive rate FPR) of a binary classifier as its discrimination threshold is varied. The AuROC curve, bounded between zero and one, is computed using a simple trapezoidal approximation. A value of one for the area means that all fixations fall on saliency hotspots of the FHM and values of 0.5 or below represent chance levels.

While the ROC is the most widely used measure of similarity it suffers from some limitations. In practice, the AuROC remains high regardless of the false negative rate, i.e., pixels proposed as fixations by the saliency models that are not in the Summary FHM. To conduct a more comprehensive evaluation, we also report the Spearman rank correlation coefficient (CC) between the Summary FHM and the maps derived from the two saliency models. The CC measures monotonic relationships between the two images and is much more sensitive than the AuROC to the false negative rate.

The statistical comparison between the prediction values of the different saliency maps across image categories was based on the Kruskall–Wallis test (non-parametric ANOVA).

## Results

### Cells in the amygdala modulate their responses by eye movements

**Figure 2:**
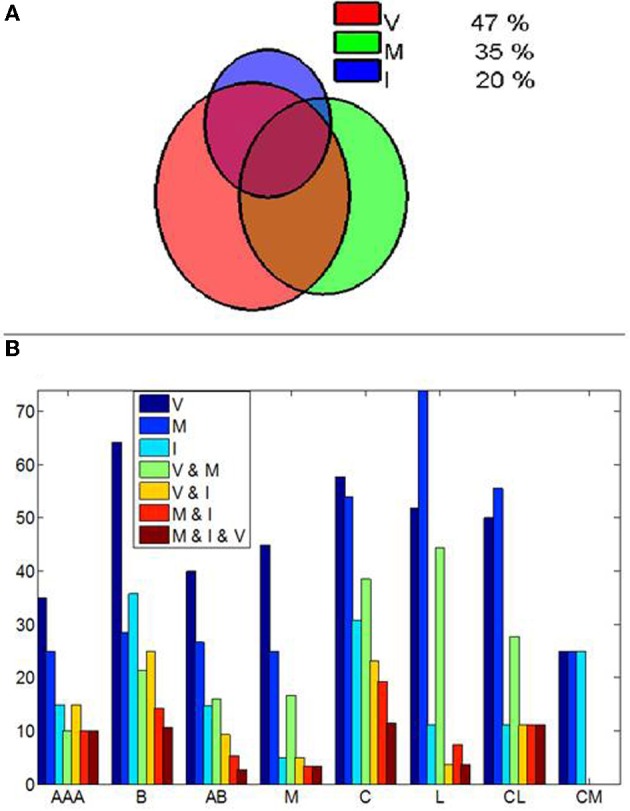
**Cells in the amygdala modulate their responses by eye movements. (A)** Venn diagram illustrating the proportion of amygdala cells showing visuo (V), motor (M) and Image (I) selective modulation. A cell showing motor selective modulation is defined as a cell which significantly modify (*p* < 0.01, Mann–Whitney *U*-test) its mean discharge rate between the saccades and fixation periods detected from fixspot presentation to the end of image viewing. **(B)** Interamygdalar distribution of cells as a function of the anatomical nuclei. Nuclei were split into: AAA, Accessory amygdaloid area; B, Basal nuclei; AB, Accessory basal division; M, medial division; C, Central division; L, Lateral Division; CL, Centrolateral division; CM, Central medial division. Note that cells showing selectivity in two or more categories are indicate by the and symbol, i.e., V and M indicates cells showing simultaneous visual and motor modulations in firing rate. While visual cells are dominant in the medial, basal, and accessory basal nuclei, motor-related effects are dominant in the lateral nuclei and the lateral division of the central amygdala. The proportion of cells with visual and motor modulation is similar in the central and medial division of the central amygdala. The combination of visuo-motor effects is also dominant in the central, and centrolateral divisions, the lateral nucli and the medial nuclei. Cells showing combined effects are indicated by the respective letters.

Cells were categorized as visually responsive, motor and visuo-motor. Visually responsive cells included cells that varied firing in response to the fixspot, the image, both or exclusively to some particular image categories (image selective). Motor cells significantly varied firing rates between saccades and fixations but exhibited no visual responses. Visuo-motor cells displayed both, significant visual responses and significant modulations in firing during saccades or fixations. The results, summarized in Figure 2A, demonstrate that a significant proportion of amygdala cells are not purely visually responsive cells. Figure 2B depicts the by nuclei distribution of responses.

Firing pattern properties and visual selectivity have been previously described for this dataset in (Mosher et al., 2010) and (Gothard et al., 2007). According to these previous results, cells in the amygdala exhibit complex visual responses to multiple categories of stimuli. Visual responses are characterized by modulations in (Gothard et al., 2007): (1) magnitude of firing rate change, (2) polarity (inhibitory vs excitatory), and (3) timing (phasic vs. tonic). Differential responses in firing rates to emotional faces as compared to neutral faces or to other non-face stimuli were found in a subset of the visual cells showing selective responses to faces. The most common modulations in firing were observed between faces and non-faces or to the onset of the fixspot. Yet, many of the cells showed selectivity for novel stimuli rather than for emotionally laden monkey faces. Considering the extensive analysis of visual responses previously reported for this dataset we focus here on the motor aspects of the responses and their link to eye movements.

The statistical comparisons of the mean firing rates over the whole task, i.e., from fixspot presentation to the end of image viewing, revealed significant changes (*p* < 0.01, Mann–Whitney *U*-tests) in the discharge rates between saccade and fixation periods for 53 (20%) of the cells. Half of these cells (27/53) showed no visual responses to the image or the fixspot. Consequently, a significant proportion of amygdala cells modulate their responses by eye movements.

**Figure 3:**
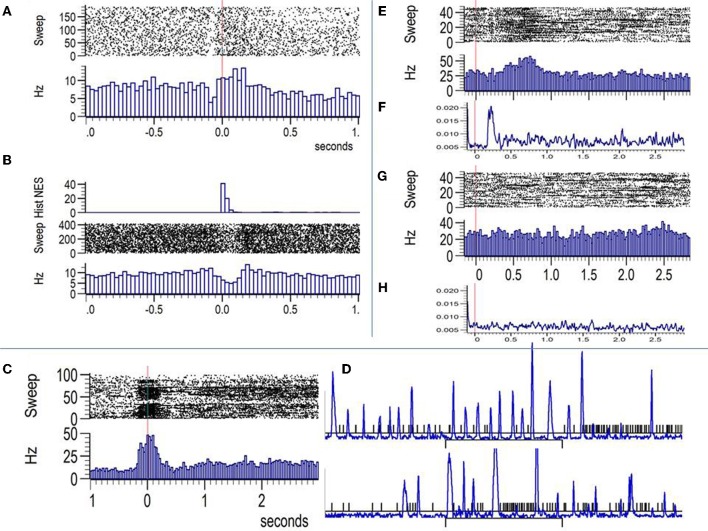
**Examples of firing rate modulations by eye movements in the amygdala. (A)** Raster plot and PSTH (Hz) aligned by the onset of the images (vertical red line). The cell shows no image selectivity but a slight increase in firing after image onset. Note the significant decrease in firing that precedes by 100ms the onset of the image. **(B)** Responses of the same cell aligned by saccades. The top inset shows the histogram of the eye speed. Note that the decrease in firing coincides with periods of increased eye speed (saccades) and a modest yet significant increase in firing is observed during fixations just before and after saccades. **(C)** A cell placed in the Centromedial nuclei showing bursts during the fixation period just before image onset and the first 100ms after image presentation. **(D)** The eye speed (blue) and time stamps for the spikes (black) lines for some representative trials of the same cell show the paucity in firing during saccades and the presence of bursts during some of the fixations. **(E** and **G)** Raster plots (top) and PSTHs (bottom) for a face selective cell aligned by image onset. **(E)** shows the response to threatening faces and **(G)** the response to non-face stimuli. Panels **(F)** and **(H)** show the mean eye speed averaged across all repetitions of threatening faces **(F)** and non-faces **(H)**. Both, firing rates and eye speed are different across TH faces and non-faces indicating early saccades in responses to TH faces that are well aligned across trials. Bursts are seen for the same cell during fixation periods following saccades.

An example of a cell classed as visuo-motor according to previous analysis is shown in panels **A** and **B** of Figure 3. In **3A** the firing rate is aligned by the onset of the image indicated by the vertical red line. Note that the cell shows no obvious response to the fixspot but significantly decrease firing during the orienting saccade to the fixspot that precedes the image onset. Firing rate increases significantly between 100–200ms after image onset. Panel **B** shows the raster and histogram of the same cell aligned by the onset of saccades detected in a 5 s window surrounding image onset (three seconds after). This period includes orienting saccades to the fixspot and saccades during image viewing. The histogram of the eye speed normalized by its maximum is shown on top of **3B**. Note the significant reduction in firing during saccades and the slight still significant increase in firing during the fixations taking place before and after saccades.

Figure 3C depicts another example of commonly observed visuo-motor modulation of firing in amygdala cells. Firing is significantly increased during the fixation period extending from the end of the orienting saccade to the fixspot to around 150ms after image onset. The cell shows no image selectivity. As shown in 3D, where the eye speed (blue) is overlaid on the spike time stamps (black), the presence of bursts during periods of sustained fixation is common during image viewing.

Of particular relevance to the interpretation of firing patterns of amygdala cells to the different categories of visual stimuli is the example illustrated in panels **E–H** of Figure 3. Panels **E** and **G** show the raster and PSTH plots aligned by the onset (vertical red line) of threatening faces and non-faces, respectively. Below the PSTHs we show (panels **F** and **H**) the eye speed averaged across the same trials. The firing rate significantly increases for the threatening faces while no change is observed for the non-face stimuli. However, the eye speed traces are also significantly different with a clear increase in eye movements between 150–350ms after image onset. This does not implies that saccades are absent for non-face stimuli but rather than they are not aligned across the image repetitions. For this session and animal, saccades for threatening faces are initiated for most of the trials within a close temporal window that shortly follows the onset of the face. Saccades are followed by long fixations reflected in the firing pattern of the cell by bursts of APs. Consequently, eye movements are a confounding factor for the interpretation of the image selectivity patterns of some cells in the amygdala that might be related to the speed or the position of the eyes during the viewing of the image.

### Scanpaths during free viewing of natural images

**Figure 4:**
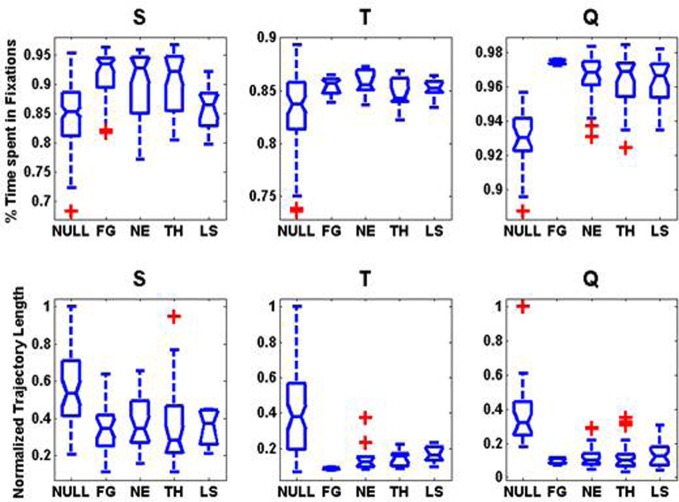
**Regardless of the emotional expression, scanpaths over faces are highly stereotyped: boxplots on the statistics on the scanpaths as a function of image category for each monkey. First Row:** Distribution of the percentage of total image viewing time spent in fixation. **Second Row:** Normalized Trajectory Length: perimeter of the convex hull enclosing all the fixations detected. For each monkey, we divided the trajectory lengths by the maximum perimeter observed within the NULL (Non-faces) category.

Regardless of the emotional expression, scanpaths over faces were highly stereotyped (Figure 4). Coinciding with previous studies (Guo et al., 2006), the percentage of time spent in fixations over faces was significantly longer than in non-faces for all three monkeys (Kruskal–Wallis test, non-parametric one-way ANOVA, *p* = 0.000002, *p* = 0.03, *p* = 0.007) with most of the time being spent into fixations of the eyes or mouth. We observed some deviations of this pattern as a function of the identity of the viewer and the gaze of the viewed. The normalized trajectory length (perimeters of the regions enclosing all fixations) were significantly larger for non-faces than for faces (Kruskal–Wallis test, all three *p*-values <1.0e-10) indicating a much larger scanpaths' variability.

Interestingly, differences in the scanpaths across facial expression were very small (Figure 4). We found little significant differences in the normalized trajectory length across expressions. In monkey T, the normalized trajectory length was significantly shorter (*p* = 0.024, *Kruskal–Wallis*) for FG faces than for other categories. The time spent in fixations was significantly longer for fearful faces than for other expressions in monkey *Q* (*p* = 0.039, *Kruskal–Wallis*). Interestingly, for monkey S who is the dominant male, the time spent in fixations for appeasing faces expressions was significantly shorter (*p* = 0.041, *Kruskal–Wallis*) than for other expressions. The lack of consistent significant differences across expressions does not however imply that the scanpaths are identical. It just indicates that all faces received comparable scanning times although the most frequently fixated facial features might have differed. Important differences across the viewers have been already described for these animals (Mosher et al., 2011) during scanning of movies.

### Spatially coincident burst firing in amygdala cells as a mechanism to indicate saliency

**Figure 5:**
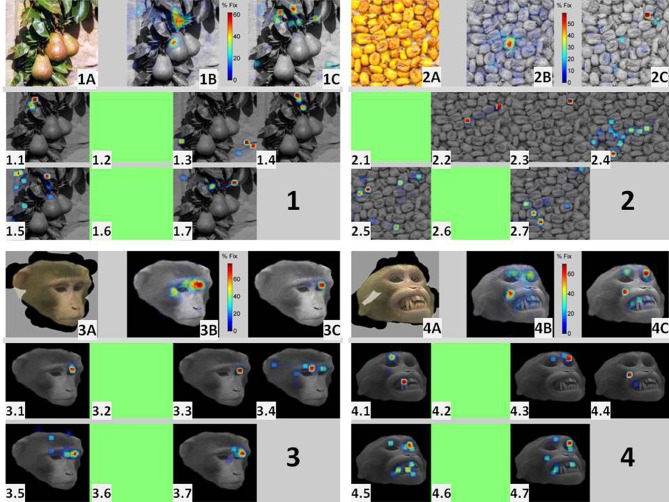
**Ensemble bursting in the amygdala of primates as a neural mechanism to signal saliency: the figure is divided into four panels, each summarizing the behavioral (eye movements) and electrophysiological responses to repeated presentations of four different images (two faces and two non-faces) within a single session.** Each one of the four images was presented a minimum of 20 times (in pseudo-random order) for 3 s. Within each panel, the upper leftmost insets (**1A, 2A, 3A, 4A**) depict the masked image obtained by veiling (in gray) pixels that were never visited by the eyes during the repeated presentations of the same image. The middle topmost insets (**1B, 2B, 3B, 4B**) represent the Summary Fixation HotSpot maps, obtained for each image by averaging all the trial-unique scanpaths. They indicate the percentage of total viewing time that the pixel was selected as a target for fixation across all presentations of the same image. The seven lower insets (1.1,…,1.7; 2.1,…,2.7; 3.1,…,3.7; 4.1,…,4.7), depict the 2D histogram of bursts for each of the seven simultaneously recorded cells in this session. A green inset indicates that the cell fired no bursts for the image. The upper rightmost insets (**1C, 2C, 3C, 4C**) depict the image obtained after adding the seven individual burst histograms.

Figure 5 illustrates the existence of coincident burst firing over salient scene locations for one experimental session where seven neurons were isolated. The figure is divided into four panels, each summarizing the behavioral (eye movements) and electrophysiological responses to repeated presentations of four different images (two faces and two non-faces). All four images were presented at least 20 times (in pseudo-random order) for 3 s. Within each panel, the upper leftmost insets (1A, 2A, 3A, 4A) depict the masked image obtained by veiling (in gray) pixels that were never visited by the eyes during the repeated presentations of the image. The middle topmost insets (1B, 2B, 3B, 4B) represent the Summary FHMs, obtained for each image by averaging all the trial-unique scanpaths. They indicate the percentage of total viewing time that the pixel was selected as a target for fixation across all presentations. The seven lower insets (1.1,…, 1.7; 2.1,…,2.7; 3.1,…,3.7; 4.1,…,4.7), depict the 2D histogram of bursts for each of the seven simultaneously recorded cells in this session. For building the histogram, 150 bins were taken along the horizontal and vertical directions of the original image (300 by 300 pixels). Green insets indicate cells firing no bursts for the image. The upper rightmost insets (1C, 2C, 3C, 4C) depict the summary histogram obtained by adding the seven individual burst histograms.

The masked images (insets A) in Figure 5 illustrate some differences in scanning faces and non-faces. Despite multiple presentations, the area of visited pixels for faces (transparent area) is practically restricted to the facial contour. In contrast, pixels in the non-face images were visited at least once over the whole session. Noteworthy, spatially restricted scanning patterns were also observed for compact non-face objects (see Figure 8). This picture highlights a source for the significantly larger variability in scanning non-faces, i.e., the scarcity of visits to the background. The Summary FHM (panels B) illustrates another source of variability. The most repeated fixation spot for the two non-faces is the central spot where the eyes fixate “waiting” for image presentation. In contrast, for faces, preferred fixations typically encompassed the eyes and/or the mouth area.

The seven burst histograms reveal interesting differences in the responses of cells to face and non-face stimuli. First, five out the seven recorded cells fired bursts for both, faces and non-faces. It is therefore impossible to attribute the presence of bursts in this population of cells to a simple selectivity for facial stimuli (Gothard et al., 2007). Second, burst histograms for non-faces (panels 1 and 2) lack a consistent pattern over the ensemble of cells. However, for faces, bursts consistently clustered around some areas (e.g., the eyes or mouth) across the cells. The fact that the same five cells fired bursts for non-faces indicate that they cannot be considered as purely “eye” or “mouth” cells (Rutishauser et al., 2011). For faces, the comparison between the FHMs and the combination of burst histograms over the ensemble of cells (i.e., 3B vs. 3C and 4B vs. 4C) reveal striking similarities which are absent for non-faces (i.e., 1B vs. 1C and 2B vs. 2C). Since perceptually salient elements of the visual scene, as detected on the Summary FHM, are known to attract longer visual exploration (Henderson and Hollingworth, 1999) then the similarity between the FHM and the bursts histograms is compatible with the existence of an ensemble bursting coding mechanism within the amygdala signaling the saliency of specific aspects within the visual scene.

Could peri-saccadic modulation of neuronal responses combined with longer fixations on salient targets be the cause—rather than the consequence—of the increased ensemble bursting observed for cells in the amygdala? Indeed, we found that 20% of the amygdala cells modulate their firing according to the saccade/fixation patterns. Since the viewer dwell longer on salient parts of the image and the discharge rates of some cells might increase during fixations, the probability of detecting bursts at pixels that are fixated longer increases. Importantly, if the mere increase in fixation times for faces enhances the firing rates of cells, then the repeated reports about face selective cells within the amygdala would require further examination. It is therefore essential to clarify this issue as most previous studies ignored fixation duration as a potential explanatory variable for face selectivity.

If ensemble bursting within the amygdala intervenes in signaling saliency then bursts firing should be a function of the spatial position of the eyes over the image irrespective of the eye speed. Indeed, if the amygdala participates in selecting salient targets for detailed scrutiny we should observe bursts when the eyes select the targets (saccades), or fixate salient parts of the images. If on the contrary, there is peri-saccadic neuronal suppression in the amygdala and the increased duration of fixations over faces trivially causes the enhancement in firing, then bursts should be absent for saccades and their number and surprise should increase with fixation duration.

**Figure 6:**
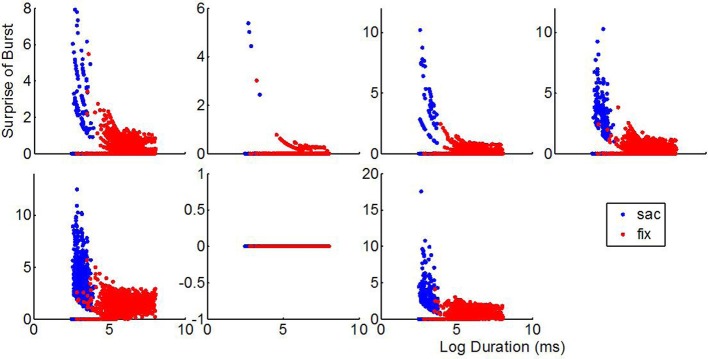
**The higher firing and surprise of bursts during fixations with respect to saccades is not due to fixations lasting longer.** Surprise of the bursts (ordinates) as a function of the duration of the saccades (blue dots) or fixations (red dots). Each inset corresponds to one of the cells shown in Figure 5 using an identical ordering. Note that bursts are absent for a large proportion of saccades and fixations regardless their duration. Indeed, the most surprising bursts are observed for relatively short saccades rather than for the longer fixations. In combination with Figure 5, these plots indicate that firing of bursts is a function of the spatial position of the eyes over the image (spatial selectivity) rather than a mere consequence of fixations.

**Figure 7:**
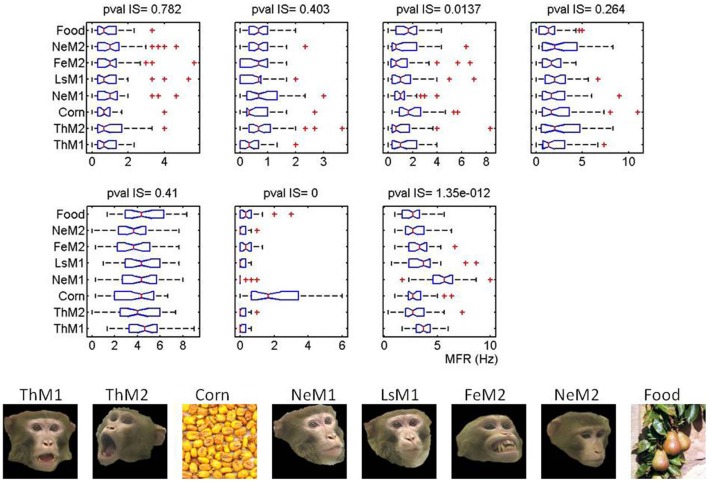
**Burst firing over salient locations is independent on the individual image selectivity for each cell.** Image selectivity for the seven cells shown in Figure 5. In the bottom panel we depict the images seen by the monkeys at this session. On top, we present the boxplots summarizing the distribution of firing rates as a function of the image. Cells are shown in the same order as in the main text. On top of each cell we give the pvalues obtained for the multiple comparisons based on the Kruskal–Wallis test (non-parametric version of ANOVA). Note that Fe in this picture stands for Fear Grimace (FG).

As illustrated in Figure 6, for the same cells as in Figure 5, bursts with high surprise appear during both, saccades and fixations. Bursts with the higher surprise values indeed happen during saccades of relatively short durations rather than during the longer fixations. Moreover, bursts are not fired during a significant proportion of fixations and saccades regardless their duration. A negative significant (*p* < 0.01) correlation between the surprise of the bursts fired during fixations and fixation duration was found for five out the six cells firing bursts. For the other cell this correlation was not significant (*p* = 0.067). These results rule out the possibility of trivially observing more bursts at certain parts of the images as a consequence of increased fixations and neuronal saccadic suppression. Curiously, while the six cells emitting bursts differ in their visual selectivity (Figure 7) they all share in common the property of firing bursts during either saccades or fixations occurring at parts of the image receiving the longest and more repeated fixations, i.e., the most behaviorally salient features. Consequently, results at the single session level support ensemble bursting in the primates' amygdala as saliency signaling mechanism.

### Ensemble bursting in the amygdala accurately predicts preferred fixations: analysis across sessions and animals

Previous results illustrate how cells in the amygdala can modulate their firing patterns by the speed (saccade or fixation) or the position of the eyes within an image. Accounting for all these factors requires an analysis approach that goes beyond the conventional raster and PSTH plots. As the scanpaths vary across repetitions of the same image and saccades/fixations can start or end at different locations or be initiated at different times it becomes hard to find the correct alignment to build the PSTHs. In addition, visual responses in the amygdala are complex. The same cell can increase the firing rate in response to the fixspot but decrease it during image presentation (Mosher et al., 2010). As already shown (Mosher et al., 2011), scanpaths vary as a function of the viewed image but also as a function of the identity of the viewer. Yet, we often observed during the visual inspection of the raw traces that cells showing motor selectivity often fired bursts of APs during some of the fixations. As shown in previous section bursts often coincide with frequently fixed (salient) aspects of the images and ensemble bursting has been previously described as a saliency signaling mechanism. We therefore reasoned that if ensemble bursting within the amygdala is the mechanism signaling salient targets then the bursts should exhibit spatial selectivity—a sort of saliency map—that coincides with pixels in the images that were repeatedly fixated across multiple image presentations or for longer periods. This rationale was followed to extend results of the previous section to all the recording sessions in the three animals as described below.

A total of 46 sessions (15 for monkey Q, 26 for S, and 5 for T) were analyzed. Bursts were detected for nearly 70% of the sessions (31/46) comprising 3059 image presentations (1495 faces and 1564 non-faces). A session was considered as suitable for analysis if at least two of the cells displayed bursts during the three seconds of image presentation. The mean number of bursts detected per second during image viewing across the population was 0.32 (±0.06 SE) with a mean burst duration of 72ms (±16). The percentage of spikes in bursts was 37% (±2) and the mean spikes in burst 8.3 (±0.46). Bursts were more common in the centromedial and centrolateral divisions of the amygdala. Summary results in Figure 10 are then based on neural/behavioral data from 22 out the initial 46 sessions (48%) including 93 cells (38% of the total number of cells) for which at least two of the recorded cells fired bursts irrespective of their selectivity.

**Figure 8:**
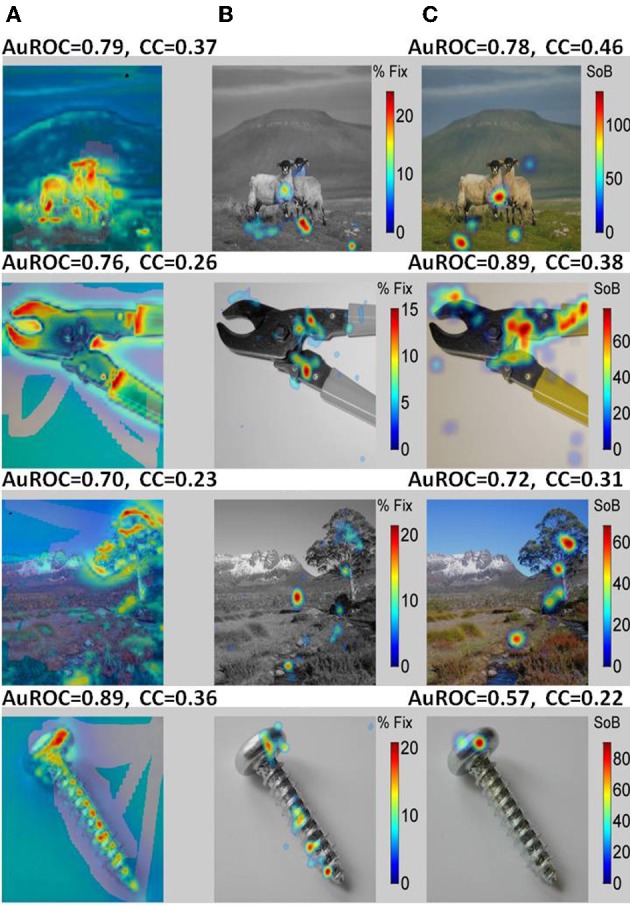
**A bottom-up saliency map (VSM) is a good predictor of fixations on natural images or objects. (A)** Examples of the natural images seen by the monkeys with VSM overlaid on top. The VSM is computed from the image features according to Itti and Koch saliency map model. **(B)** Summary Fixation HotSpot maps for multiple presentations of the same image thresholded to emphasize pixels receiving the longer fixations across image repetitions. The map is constructed from averaging and smoothing the scanpaths executed by the viewer over each image presentation and indicates places in the image that were behaviorally salient for the viewer and hence repeatedly visited. **(C)** Amygdala Saliency model (ASM) generated from the firing of ensembles of cells in the amygdala. Colors in the image represent the surprise of the bursts of action potentials detected from small ensemble of cells corrected by the time spent in visiting the corresponding pixel. The numbers above the two models represent: (1) the AuROC that gives a scalar similarity measure between a model and the actual fixation map. A value of one for the area means that all fixations fall on saliency hot spots of the maps and values of 0.5 or below represent chance levels. (2) The Spearman Rank correlation coefficient (CC) between the corresponding saliency model and the Summary Fixation HotSpot map. Note that the AuROC for the VSM **(A)** is higher than 0.7 for all the images shown. This implies that bottom-up visual saliency directly detected from the image features predicts a significant proportion of fixations on non-faces images.

**Figure 9:**
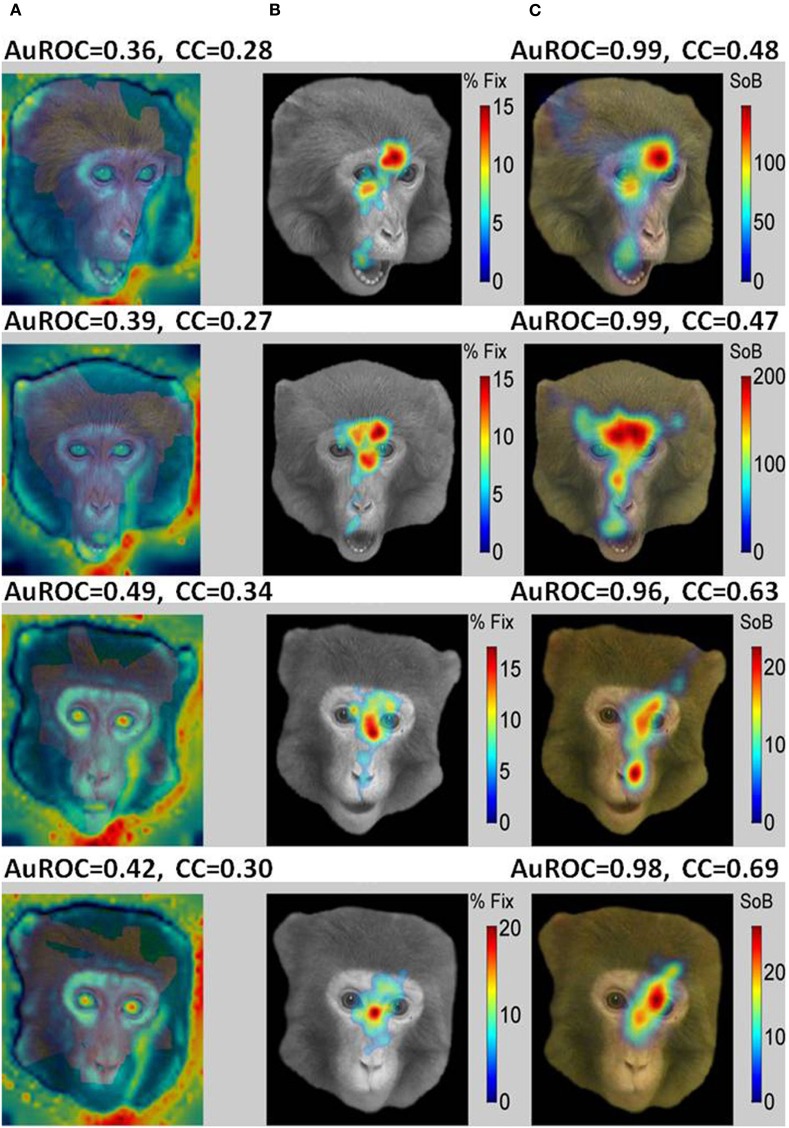
**A saliency model generated from the burst firing of ensemble of cells in the primates amygdala (ASM) is an excellent predictor of fixations on faces while bottom-up visual saliency (VSM) fails. (A)** Examples of faces portraying different facial expressions seen by the monkeys with VSM overlaid on top. **(B)** Summary Fixation HotSpot maps for multiple presentations of the same face emphasizing pixels that received significantly longer fixations across repetitions of the same face. **(C)** Amygdala Saliency model (ASM) generated from the firing of ensembles of cells in the amygdala. The numbers above the two models represent: (1) the value for the AuROC and the Spearman rank correlation (CC). Contrarily to the results obtained for non-faces, the AuROC for the VSM (7A) is at chance level (below 0.5). Consequently, bottom-up visual saliency is not a good predictor of fixations on faces that seems to depend on internally defined (top-down) aspects such as viewer experience and goals. In contrast, the AuROC for the ASM is higher than 0.96 for all the images shown. This indicates that for face stimuli nearly all prolonged fixations coincide with bursts fired by the ensemble of cells.

Typical examples of the presented images are shown in Figure 8 (Non-faces) and Figure 9 (Faces). The VSMs constructed by combining with equal weights color, depth, and contrast into one global measure of each image determined saliency are depicted in the first column of Figures 8 and 9 (8A, 9A). On top of the plot we give the value of the AuROC and the CC for the VSM. The summary FHMs are shown in Figures 8B and 9B. Figures 8C and 9C depict the ASM resultant from the normalized ensemble bursts across all repetitions of the image. On top we report the AuROC for the prediction of the summary FHM and the Spearman rank correlation coefficient (CC) between the ASM and the summary FHM.

For the examples shown, the VSM is a bad predictor of fixations over faces with values below the 0.5 chance level. On the other hand, predictions on objects and landscapes obtained from the VSM are above chance and relatively good. The ASM is an excellent predictor of fixation on faces and is above chance (AuROC curve higher than 0.5) for non-faces as well. The high values of the AuROC for all categories of images and particularly for faces indicate that a majority of the most frequently fixated pixels coincide with places where bursts were detected within the sampled ensemble of cells.

**Figure 10:**
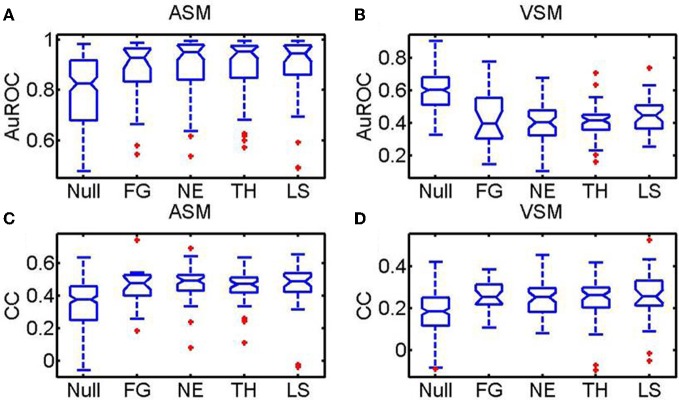
**Statistics over the whole population of faces and non-face images (NULL) pooled across monkeys using the AuROC and the 2D correlation coefficient as figures of merit.** Faces are divided according to the four facial expressions: (Ne, Neutral; Th: Threatening; LS: Appeasing expression; FG: Fear). Tukey box-plots of the area under the ROC curve (AuROC) and the correlation coefficient (CC) are depicted on each row. The box-plots in **(A)** and **(C)** are obtained for the amygdala saliency map (ASM) and the box-plots in **(B)** and **(D)** for the visual saliency map (VSM). According to the AuROC values shown in the upper insets (**A** and **B**) bottom-up saliency, as defined by Itti and Koch, is a relatively good predictor of fixations on landscapes and objects but it is systematically below chance for faces. Ensemble bursting in the primates' amygdala is an excellent predictor of fixations over faces regardless of facial expression. As happens with the AuROC, the ASM correlates (CC) significantly better (*p*_*f*_, < 0.01, Kruskall–Wallis) with the fixation map for faces than for non-faces. However, no significant differences exist in the prediction of the fixation patterns across the different facial expressions (*p*_*e*_ < 0.1, Kruskall–Wallis).

Summary statistics over all images, sessions and monkeys for the AuROC and the CC are shown in Figure 10. In the figure, faces are divided according to the portrayed emotion—(1) LS (Lip smacking, appeasing face), (2) TH (threatening), (3) NE (Neutral), (4) FG (Fear Grimace). The category NULL encompasses a broad group of non-faces images including food, landscapes, abstract pictures and objects. The two upper insets depict the summary statistics for the AuROC. The two lower insets correspond to the CCs. Predictions for the ASM are given in the left insets and for the VSM in the right insets.

For all monkeys, the VSM (10B) fails to predict (AuROC curve below 0.5) the fixations on faces regardless of the emotion portrayed but it is above chance for non-faces (NULL). The mean values of the AuROC curve obtained from the ASM are all above 0.8 (10A). Predictions on faces (mean AuROC curve higher than 0.9) are, nonetheless, significantly better than on non-faces (*p* < 0.01, non-paired, non-parametric Mann–Whitney *U*-tests). The correlation coefficients (CC, 10C and 10D) show a slightly different trend as this measure is more sensitive than the AuROC to the rate of false alarms, i.e., to fixations predicted by the model not falling on hotspot pixels within the Summary FHM. The CC values are, for both the ASM (10C) and the VSM (10D), significantly higher for faces than for non-faces. This is likely due to the inherent variability in fixation positions across repetitions for non-faces. While the correlation values were overall lower than the AuROC curve, the ASM correlates significantly better with the fixation map for faces than for non-faces objects. Mean correlation for faces typically reaches values of 0.5 which indicates a highly significant resemblance over a 300 × 300 pixels matrix. Unexpectedly, neither the AuROC curve nor the correlation revealed differences in the prediction of the fixation patterns across the different facial expressions (*p* > 0.1, non-paired, non-parametric Mann–Whitney *U*-tests).

**Figure 11:**
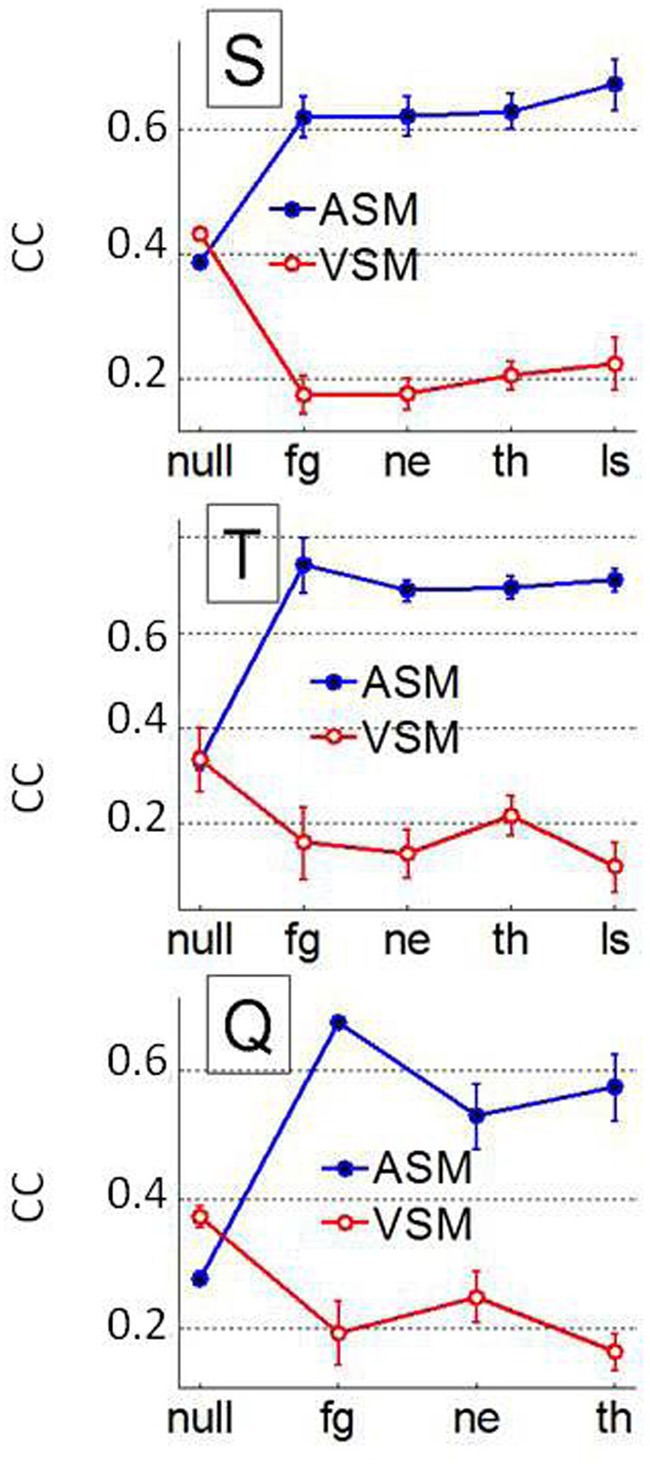
**Interindividual differences over the whole population of faces and non-face images (NULL) using the AuROC 2D correlation coefficient as figures of merit.** Mean and 95% confidence interval around the mean for the correlation coefficient (CC) between the HotFix and ASM/VSM computed for each monkey. Ensemble bursting in the primates' amygdala is a better predictor of fixations over faces than low level visual features but interindividual differences in prediction across facial expression starts to weakly correlate with interindividual differences in behavior (see Figure 4).

To assess if pooling across animals could have masked interindividual differences in the prediction of the fixation patterns we repeated previous analysis at the individual level. Patterns for both, the AuROC curve and the CC were very similar to those observed when animals were pooled, i.e., predictions were significantly higher for faces than for non-faces. The VSM also failed to predict fixations over faces at the single monkey level irrespective of facial expression. Figure 11 shows the individual mean CC and 95% confidence interval around it as a function of image category for both the ASM and the VSM. For monkey S, the CC values for the ASM are slightly (*p* = 0.2, Kruskall–Wallis) higher for LS faces than for other categories. The AuROC revealed no significant differences in prediction across facial expressions (not shown). Yet, the CC values (Figure 11) revealed some interindividual variability in the prediction of fixations based on the ASM across facial expressions. The observed differences somehow evoke the interindividual differences in scanning patterns shown in Figure 4. For monkeys Q and T, CC values are higher for fearful faces than for other categories of faces and this difference becomes significant for monkey Q (*p* = 0.024, Kruskall–Wallis) but not for monkey T (*p* = 0.1). Note that no bursts were detected in sessions where LS faces were presented to monkey Q and therefore CC or AuROC values are lacking for this facial expression. Nevertheless, even if the link between CC values and individual behavior remains statistically weak, this link between interindividual variability in fixation locations and the firing of amygdala cells deserves further investigation.

## Discussion

The summary statistics over animals and sessions support the existence of a general mechanism within the amygdala based on the coincidence of bursts across cell ensembles to signal the saliency of targets within a visual scene. The significantly better prediction observed for faces using the ASM indicates two things. First, the cells are not firing bursts for every fixation but just for fixations over relevant parts of the images. Otherwise, we would have obtained perfect predictions of the Summary FHM regardless of image category. Second, the saliency defined by the ensemble bursting appears to be more driven by the goals and experience of the viewers (top-down) than by low level image features (bottom-up). Later observation also stems from the differences in prediction across image categories observed between the ASM and the VSM.

Could a simpler analysis based on a rate code provide similar prediction results? This is very likely as a burst necessarily implies an increase in the firing rate. Yet, there are several reasons to justify our selection of bursts as the basis of a potential saliency coding mechanism. First, and more importantly, the visual inspection of the raster plots indicated that most cells with motor modulation fired bursts at several fixations irrespective of the time spent after image onset. In contrast, most purely visual cells fired shortly after image onset, decreasing firing afterwards. Therefore bursts' firing was a more stable feature in relationship to eye movements than rate coding along the whole image viewing period. Second, the complexity of visual responses in the amygdala (e.g., phasic responses) needs to be further investigated to understand better what aspects of motivation or saliency are coded when the same cell enhances firing for the fixspot and decreases it for any image. Third, ensemble bursting has been shown to provide a coding mechanism for motivation (Lin and Nicolelis, 2008) in the basal forebrain, structure that is functionally close to the amygdala (Alheid, 2003; Liberzon et al., 2003). Fourth, target detection which is a correlate of saliency is coded in the form of bursts by the supplementary or the supplementary eye field (Hanes et al., 1995).

Since our goal was to compare the predictive power of a top-down (ASM) and a bottom-up (VSM) model, in the absence of any particular a priori, we used the simplest combination of weights to build the VSM (Itti et al., 1998). Still, the VSM was efficient in predicting fixations over non-faces but failed over faces. This suggests that experience (top-down) more than visual features (bottom-up) determine spatial-temporal patterns of scanpaths over faces (Guo, 2007), at least over the early scanning periods. This is not surprising since several learned factors need to be considered before fixating gaze on, for example, the eyes. While the eyes are fairly salient in visual terms due to their contrast with the surrounding pixels—evident from the VSM images—their behavioral saliency necessarily changes as a function of the context. Under many circumstances primates avoid direct gaze contact as it can be interpreted as a threatening signal. Such contextual information cannot be inferred from visual features alone as it depends on multiple factors such as the instantaneous goals and social status of the viewer or the emotional expression and gaze of the viewed.

The properties expected for the locus of the theoretical saliency map include the following necessary conditions (Fecteau and Munoz, 2006): (1) neurons should encode visual information in a featureless manner, (2) lesions should produce deficits in attentional selection, (3) electrically stimulating these regions should facilitate the selection of objects with attention, (4) the structure should receive information from the ventral pathway. The amygdala fulfills all these properties. First, the amygdala contains cells with wide receptive fields selective for aspects such as identity, gaze, or facial expression (Gothard et al., 2007; Hoffman et al., 2007), representing them in a featureless manner (Fecteau and Munoz, 2006; Baluch and Itti, 2011). Amygdala cells encode behavioral relevance for nearly all sensory modalities, a main component of saliency, with enough flexibility to quickly adapt to the immediate goals of the observer or to changes in the external significance of the stimuli (Gallagher and Holland, 1994; Paton et al., 2006). Second, lesions to the amygdala lead to deficits in selective visual orienting in animals (Gallagher and Holland, 1994) and humans (Akiyama et al., 2007), abolish orienting to novel visual stimuli (Bagshaw et al., 1972) or to parts of faces that are typically attended (Adolphs et al., 2005). Third, electrical stimulation of the amygdala can initiate orienting responses with quick and/or anxious glancing and searching movements of the eyes and head such that the organism appears aroused and highly alert as if in expectation of something that is going to happen (Ursin, 1960; Applegate et al., 1983). Four, the amygdala is reciprocally connected with the inferior temporal cortical areas TEO and TE within the ventral visual stream (Webster et al., 1991) from which receives highly processed visual information. Consequently, although not previously acknowledged, the amygdala fulfills the necessary conditions to store a map of saliency.

Our results contribute in several ways to complete this picture on the amygdala as the locus of a saliency map (Fecteau and Munoz, 2006). First, we have provided the first electrophysiological evidence for the spatial coincidence of bursts firing across population of cells in the amygdala with the parts of the images that were prioritized by the animals while freely scanning them. Second, we have identified the ensemble bursting within the amygdala as a potential computational mechanism that—in similarity to other neural structures (Hanes et al., 1995; Lin and Nicolelis, 2008)—serves to signal saliency. Third, we have shown that single cells in the amygdala fire bursts during saccades or fixations done over salient image targets suggesting that the amygdala might be also part of the oculo-motor control network. Fourth, we have shown that ensemble bursting predicts with an excellent accuracy the fixation patterns of the monkeys over faces and a significant part of fixations over non-faces.

In summary, our findings extend the established role of the amygdala in visual orienting (Gallagher and Holland, 1994) by suggesting a computational mechanism—burst ensemble—to define where and for how long to look on the basis of an internally established model of saliency. Importantly, our observations support previous studies on the specificity of visual responses in the amygdala (Rolls et al., 1994; Gothard et al., 2007; Rutishauser et al., 2011) since suggesting the selectivity in firing as the cause, rather than the consequence, of the variability in scanning patterns between face and non-faces. As a whole, our findings support the purported role of the amygdala in defining saliency, define the ensemble bursting as a potential computational mechanisms involved and propose the amygdala as a candidate to store a saliency (Baluch and Itti, 2011; Fecteau and Munoz, 2006) map. These results help clarifying the link between observations of abnormal fixation patterns in autism or schizophrenia and structural damage to the amygdala and might help to broaden the current view on amygdala function to encompass a large number of experimental observations linking the amygdala to emotion, novelty detection, attention, and reward. Emotion and fear are important dimensions of saliency but not necessarily the only ones.

### Conflict of interest statement

The authors declare that the research was conducted in the absence of any commercial or financial relationships that could be construed as a potential conflict of interest.
